# P-953. Infection prevention certificate exam performance: ChatGPT 4 vs ChatGPT 3.5

**DOI:** 10.1093/ofid/ofae631.1143

**Published:** 2025-01-29

**Authors:** Saujanya Saravanakumar, Sureshkumar Dorairajan, Jenifer Naveen, S Shobana

**Affiliations:** Stanlry Medical College, Chennai, Tamil Nadu, India; Apollo hospital, Chennai, Chennai, Tamil Nadu, India; Madras Medical Mission, Chennai, Tamil Nadu, India; Apollo Hospital, Chennai, Tamil Nadu, India

## Abstract

**Background:**

In recent times, AI models were utilized to evaluate competitive examinations such as the US medical licensing examination and clinical case conferences. However, there was limited data on their evaluation in infection control certificate examinations, known for their inclusion of complex questions. In this study, we aimed to assess the performances of ChatGPT-4 and ChatGPT-3.5 models in handling infection control certification examinations, providing insights into their efficacy in infection prevention.

**Figure:**
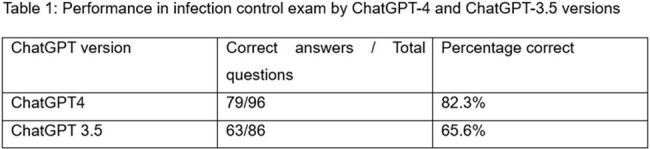

**Methods:**

This cross-sectional study, conducted by an infectious disease (ID) physician, involved an ENT resident, an ID physician assistant, and a Pharm D student. Questions were sourced from the International infection control certification examination and underwent complexity validation and answer approval by international infection prevention professionals. Each model, ChatGPT-4 and ChatGPT-3.5, received questions separately, and their answers were cross-checked with validated responses by the team. Statistical analysis employed the Chi-square test to assess performance differences. Data were collected in an excel sheet for subsequent analysis.

**Results:**

In the evaluation of 96 infection control examination questions, ChatGPT-4 outperformed ChatGPT-3.5, achieving an accuracy of 82.3% (79 out of 96 questions answered correctly) compared to ChatGPT-3.5's accuracy of 65.6% (63 out of 96) as shown in table -1 below. Statistical analysis using a Chi-square test confirmed a significant difference between the two models (p < 0.05), highlighting the advancements in ChatGPT-4's capabilities. ChatGPT-4 excelled in contextual comprehension, while ChatGPT-3.5 struggled with adaptability in managing complex queries.

**Conclusion:**

In this study, ChatGPT-4's accuracy of 82.3% exceeded that of ChatGPT-3.5, which achieved 65.6%. Future research should compare other AI models and analyse each question for contextual understanding to enhance AI models performance in infection prevention.

**Disclosures:**

**All Authors**: No reported disclosures

